# Acute, Delayed and Chronic Remote Ischemic Conditioning Is Associated with Downregulation of mTOR and Enhanced Autophagy Signaling

**DOI:** 10.1371/journal.pone.0111291

**Published:** 2014-10-27

**Authors:** Sagar Rohailla, Nadia Clarizia, Michel Sourour, Wesam Sourour, Nitai Gelber, Can Wei, Jing Li, Andrew N. Redington

**Affiliations:** Division of Cardiology, Labatt Family Heart Center, Hospital for Sick Children, University of Toronto, Ontario, Canada; Emory University, United States of America

## Abstract

**Background:**

Remote ischemic conditioning (RIC), induced by brief periods of limb ischemia has been shown to decrease acute myocardial injury and chronic responses after acute coronary syndromes. While several signaling pathways have been implicated, our understanding of the cardioprotection and its underlying mediators and mechanisms remains incomplete. In this study we examine the effect of RIC on pro-autophagy signaling as a possible mechanism of benefit.

**Methods and Results:**

We examined the role of autophagy in the acute/first window (15 minutes after RIC), delayed/second window (24 hours after RIC) and chronic (24 hours after 9 days of repeated RIC) phases of cardioprotection. C57BL/6 mice (N = 69) were allocated to each treatment phase and further stratified to receive RIC, induced by four cycles of 5 minutes of limb ischemia followed by 5 minutes of reperfusion, or control treatment consisting solely of handling without transient ischemia. The groups included, group 1 (1W control), group 2 (1W RIC), group 3 (2W control), group 4 (2W RIC), group 5 (3W control) and group 6 (3W RIC). Hearts were isolated for assessment of cardiac function and infarct size after global ischemia using a Langendorff preparation. Infarct size was reduced in all three phases of cardioprotection, in association with improvements in post-ischemic left ventricular end diastolic pressure (LVEDP) and developed pressure (LVDP) (P<0.05). The pattern of autophagy signaling varied; 1W RIC increased AMPK levels and decreased the activation of mammalian target of rapamycin (mTOR), whereas chronic RIC was associated with persistent mTOR suppression and increased levels of autophagosome proteins, LC3II/I and Atg5.

**Conclusions:**

Cardioprotection following transient ischemia exists in both the acute and delayed/chronic phases of conditioning. RIC induces pro-autophagy signaling but the pattern of responses varies depending on the phase, with the most complete portfolio of responses observed when RIC is administered chronically.

## Introduction

The additional injury incurred as a result of reperfusion after prolonged coronary ischemia currently limits clinical strategies against myocardial ischemia-reperfusion (IR) injury. Despite a greater understanding of the mechanisms underlying IR injury, interventions aimed at improving post-ischemic cardiac function have been suboptimal [1]. A promising strategy involves exposing remote tissues to brief periods of sub-lethal ischemia, which protects the myocardium against damage during and after a subsequent prolonged ischemic insult, a phenomenon termed remote ischemic conditioning (RIC) [2]. Induced by brief periods of limb ischemia and reperfusion using a blood pressure cuff or tourniquet, RIC has been rapidly translated from animal models to proof-of-principle clinical trials in acute IR syndromes [3]–[5].

RIC induced by limb ischemia is a neuro-humoral stimulus resulting in the release of circulating cytoprotective factors which induce protection against IR injury in distant organs. The mechanisms underlying RIC-induced cytoprotection have recently been reviewed in detail ([2]), but key components of cardioprotection involve stimulation of g-protein coupled receptors on the cell-surface, induction of a intracellular kinase signaling, opening of potassium-ATP (K^+^_ATP_) channels and preventing the formation of the mitochondrial transition pore (mPTP). The cardioprotection afforded by RIC is usually described as biphasic. There is an early/acute cytoprotective phase, termed the first window (1W) of protection, which essentially recapitulates the changes in intracellular kinase signaling pathways previously described for local ischemic conditioning (IC) [6]–[9]. Approximately twenty-four hours after the original stimulus, a ‘second window’ (2W) of protection emerges, that likely relates to transcriptional changes induced within the cell [10]. While the effects of the delayed response may be less robust, there is emerging clinical [11], [12] and experimental [13], [14] evidence that RIC, repeated over time (chronic RIC-3W), may have additional disease-modifying effects. For example, chronic RIC administered for 28 consecutive days in rats was associated with a reduction in post-myocardial infarction (MI) left ventricular remodeling and improved survival at 12 weeks [14].

The exact mechanisms underlying these responses remain to be delineated precisely, however there is emerging evidence for a key role of autophagy signaling in the adaptive and maladaptive responses to IR injury [15]. Indeed, several studies have highlighted a protective role of autophagy in myocardial IR injury. In one study, inhibition of mammalian target of rapamycin (mTOR), a known negative regulator of autophagy, led to a decrease in infarct size in mice [16]. A separate study using microarray analysis of mice that underwent chronic local IC, through repetitive coronary occlusion, revealed upregulation of genes associated with autophagy and the unfolded protein response [17], [18]. The possible role of RIC in autophagy modulation remains unknown. We therefore studied in-vivo myocardial signaling and assessed cardioprotection in a mouse Langendorff isolated heart model, to examine autophagy responses in the acute, delayed and chronic phases of cardioprotection afforded by RIC.

## Materials and Methods

All animal protocols were approved by the Animal Care and Use Committee of the Hospital for Sick Children in Toronto and conformed to the *Guide for the Care and Use of Laboratory Animals* published by the National Institutes of Health (NIH publication No. 85-23, revised 1996).

### Induction of remote ischemic conditioning (RIC)

RIC was induced by four cycles of 5 minutes of limb ischemia (by tourniquet tightened at the inguinal level) followed by 5 minutes of reperfusion as previously described [6]. Distal limb pallor was observed during occlusion, followed rapidly by brisk reactive hyperemia during reperfusion.

### Experimental design

C57/BL6 male mice (8–10 weeks old, N = 69) were habituated to handling by experimenters for ten minutes a day two days prior to induction of RIC. Control animals were treated identically with the tourniquet applied around the leg but not tightened and were handled for the same duration as RIC.

### Myocardial autophagy signaling

Mice were divided in to three treatment categories based on conditioning modality which include: acute/first window (1W), delayed/second window (2W) and chronic/third window (3W). Within each category, mice were further stratified to receive RIC according to the protocol described or control treatment consisting solely of handling without applying transient ischemia to the limb. The groups include, group 1 (1W control), group 2 (1W RIC), group 3 (2W control), group 4 (2W RIC), group 5 (3W control) and group 6 (3W RIC).

**Figure 1:**
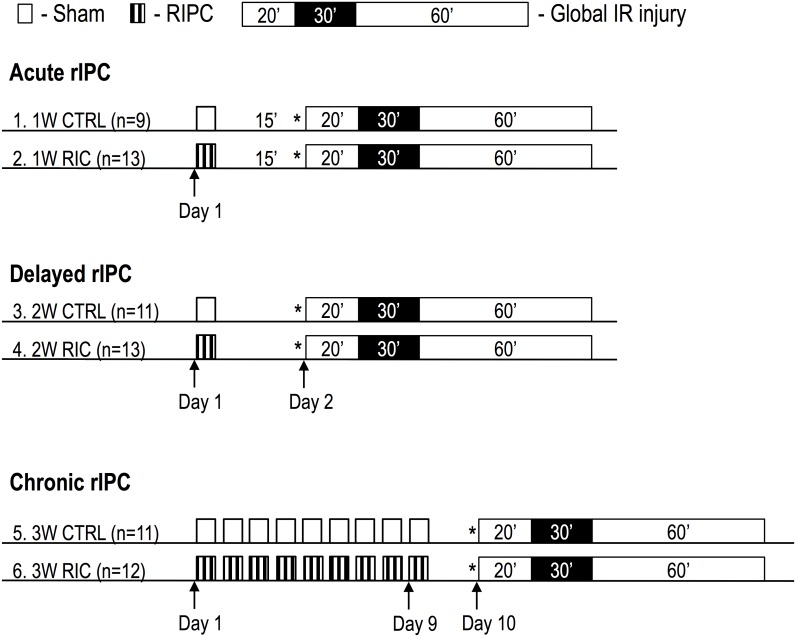
Schematic summary of RIC treatment groups and experimental protocol. Control groups 1, 3 and 5 were left untreated and underwent handling alone without RIC. Treatment groups 2, 4, and 6 underwent RIC via four cycles of 5 min of femoral artery occlusion, followed by 5 min of reperfusion. Following the treatment protocol, mouse hearts were removed (denoted by *), mounted on a isovolumic Langendorff perfusion apparatus, and subjected to 20 min of equilibration, 30 min of no-flow global ischemia and 60 min of reperfusion. The infarcted area was visualized using TTC following global ischemia in each group.

At the end of each experiment, mice were anesthetized with pentobarbital (60 mg/kg via an intra-peritoneal injection) and their hearts were isolated for further study of protein expression using western blot analysis or cardiac function using a Langendorff isolated heart model of global ischemia. To assess the first window of RIC, mice were sacrificed fifteen minutes after the end of the RIC or handling. Mice in the second window groups underwent a similar protocol but were sacrificed 24 hours after handling or RIC. To assess the chronic effects of repeated RIC, mice underwent 9 days of repeated limb ischemia or handling and were sacrificed twenty-four hours after the last day of treatment. (See figure 1 for experimental protocol).

### Immunoblotting

Following RIC or control treatment, mouse hearts were excised and cut transversely to separate the atrium and ventricle. Ventricular tissue was then used for western blot experiments. Stored heart tissue was homogenized using lysis buffer (1% NP40, 150 mM NaCl, 1 mM EGTA, 1 mM EDTA, 2.5 mM Na_4_O_7_P_2_, 1 mM β-glycerolphosphate, 1 mM Na_3_VO_4_ and a cocktail of protease inhibitors (Roche)). The homogenate was centrifuged at 10,000×*g* at 4°C for 30 min to obtain cytosolic protein. An equal amount of protein (30 µg protein) from each sample was separated by 10% SDS–polyacrylamide gels and transferred onto nitrocellulose membranes (Bio-Rad). Membranes were incubated with Atg5 (catalog #8540 – Cell Signaling Tech.), phosphor-mTOR (Ser2481) (catalog #2974 – Cell Signaling Tech.), beclin-1 (catalog #3495 – Cell Signaling Tech.), p62 (catalog #5114 – Cell Signaling Tech.), LC3I/II (NB100-2331 – Novus Biologicals), phospho-AMPK (Thr 172) (catalog #2531 – Cell Signaling Tech.), cathepsin L (catalog #AF1515 – R&D Systems) or GAPDH (catalog #G8795 - Sigma-Aldrich Inc.) overnight at 4°C. The membranes were then washed and subsequently incubated with peroxidase-conjugated secondary antibody (Santa Cruz, CA) and detected with the ECL plus Detection Kit (Amersham, Piscataway, NJ). Immunoblots were scanned using an Odyssey LI-COR and quantified using Image Studio (Ver 2.1).

### Assessment of cardioprotection in mouse Langendorff preparation using global ischemia/reperfusion injury

Mice from each study group were assessed for cardiac function following global ischemia-reperfusion injury. After completion of experimental intervention, mice received heparin (200 IU, i.p. Sigma) and were anesthetized with pentobarbital (60 mg/kg, i.p. Ceva Sante animale) and then intubated and ventilated. Hearts were rapidly excised by bilateral thoracotomy, placed in ice-cold buffer and the aorta cannulated with a 20-gauge metal cannula. Isolated hearts were mounted on the Langendorff perfusion apparatus (Radnoti Technologies Inc., Monrovia, CA, USA), and perfused under non-recirculating conditions at a constant pressure of 80 mmHg with 37°C Krebs–Henseleit buffer (KHB) (consisting of the following in mmol/L: NaCl 120.0, NaHCO_3_ 25.0, KCl 4.7, MgSO_4_ 1.2, KH_2_PO_4_ 1.2, CaCl_2_ 2.5, EDTA 0.5 and glucose 15). The left atrial appendage was removed, and a balloon, made with saran wrap and PE60 polyethylene tubing, was inserted into left ventricular (LV) through the mitral valve and was connected to a pressure transducer. The balloon was inflated with water to adjust left ventricular end-diastolic pressure (LVEDP) to 7–10 mmHg at the beginning of the experiment and the volume kept constant for the duration of the study. After a 20 min stabilization period, subjected to 30 min of no-flow global ischemia followed by 60 min of reperfusion. Hemodynamic measurements, including heart rate (HR), peak left ventricular pressure (LVP), maximum rate of contraction (+dP/dtmax), maximum rate of relaxation (−dP/dtmin), and LVEDP will be recorded on a data acquisition system (PowerLab, ADInstruments) throughout the procedure. The LV developed pressure (LVDP) was calculated as the difference between the systolic and end-diastolic LV pressures.

### Measurement of infarct size

After reperfusion the hearts were weighed and frozen at −80°C. The frozen heart was transversely cut into six 1-mm thick slices using a Mouse Heart Slicer Matrix (Zivic Instruments) which were stained with 1.25% 2,3,5-triphenyltetrazolium chloride (TTC) in 200 mM Tris/HCL solution (pH 7.4) for 15 min in a 37°C water bath. After staining, heart slices were fixed for 2–4 hours in 10% neutral buffered formaldehyde. Both sides of each slice was then photographed at 1200 DPI resolution using a computer scanner (CanoScan 4400F). Images were processed with Adobe Photoshop CS2 software to measure infarct size and left-ventricle area using automated planimetry. Viable myocardium stains red due to the reaction of tetrazolium salts with NADH and dehydrogenase enzymes while infarcted tissue, that does not possess enzymes, appears pale. Infarct sizes of each slice were expressed as the percentage of the total left ventricle area.

### Statistical Analysis

The analysis method for array data is discussed previously. For all other comparisons, statistical significance was determined using one-way ANOVA, followed by post hoc testing (Newman–Keuls) where appropriate. Values of P≤0.05 were considered statistically significant. Data are shown as mean ± S.E. (standard error).

## Results

### Chronic RIC reduces infarct size following global ischemia

**Figure 2:**
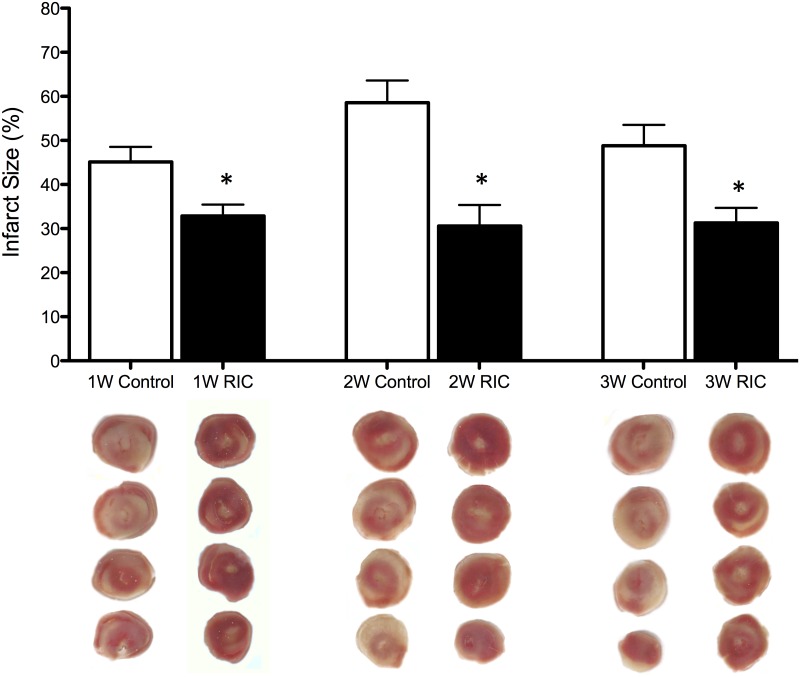
RIC reduces heart infarct size in the acute, delayed and chronic phases of conditioning. (A) Infarct size expressed as percentage of left ventricle area for each of the treatment groups. Values are means ± S.E.M. An (*) denotes a statistically significant difference compared to controls (P<0.05). (B) Representative cross-sections of mouse hearts from each of the treatment groups after I/R and staining with TTC to visualize the infarcted area.

Myocardial infarct size was assessed by TTC staining (figure 2). As previously shown in other studies we observed a significant (P<0.05) reduction in 1W RIC treated mice (32.9±2.6%). compared with 1W controls (45.1±3.4%) We also observed protection against infarction after delayed and chronic preconditioning. Both 2W RIC (30.6±5.1%) and 3W RIC (31.3±3.4%) treated mice had significantly (P<0.05) reduced infarct sizes compared to their respective control groups, 2W control (58.6±5.0%) and 3W control (48.8±4.8%).

### Chronic RIC protects post-ischemic left ventricular function

**Figure 3:**
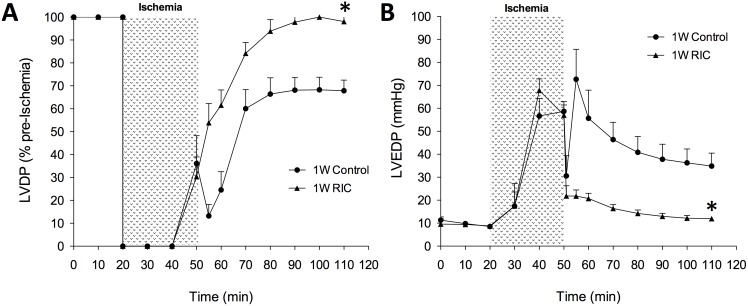
Acute, first window RIC improves post-ischemic cardiac performance in isolated perfused hearts. Representative LVDP (A) and LVEDP (B) tracings are shown from 1W control and RIC groups. Left ventricular developed pressure (LVDP) is expressed as a percentage of pre-ischemic baseline values and left ventricular end diastolic pressure (LVEDP) is expressed in mm Hg. Values are expressed as means ± S.E.M. An (*) denotes a statistically significant difference (P<0.05) compared to control groups after 60 minutes of reperfusion.

We also examined the effects of acute and chronic RIC on left ventricular function using an isolated Langendorff heart model of global ischemia. As previously shown, acute 1W RIC ameliorates the effects of ischemia-reperfusion injury on the heart with improvements in LVDP and LVEDP. Measured as a percentage of baseline function, we found that after 60 minutes of reperfusion, there was a significantly (P<0.05) greater level of recovery in developed pressure (figure 3A) in 1W RIC mice compared to controls (98.0±4.9% vs. 67.9±4.7%). Consistent with previous reports we also observed marked differences in diastolic recovery after RIC during the first window of treatment (figure 3B), as 1W RIC mice had a significantly (P<0.001) attenuated increase in diastolic pressure (LVEDP) compared to 1W controls (11.9±1.1 mmHg vs. 34.9±5.7 mmHg) after 60 minutes of reperfusion.

**Figure 4:**
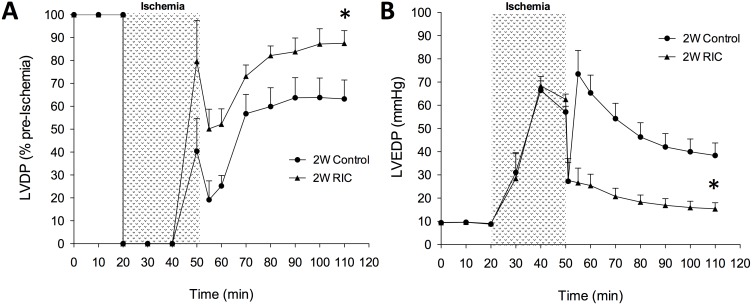
Delayed, second window RIC improves post-ischemic cardiac performance in isolated perfused hearts. Representative LVDP (A) and LVEDP (B) tracings are shown from 2W control and RIC groups. Left ventricular developed pressure (LVDP) is expressed as a percentage of pre-ischemic baseline values and left ventricular end diastolic pressure (LVEDP) is expressed in mm Hg. Values are expressed as means ± S.E.M. An (*) denotes a statistically significant difference (P<0.05) compared to control groups after 60 minutes of reperfusion.

**Figure 5:**
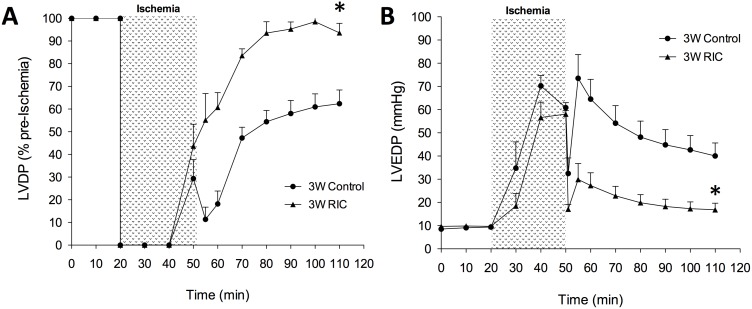
Chronic, third window RIC improves post-ischemic cardiac performance in isolated perfused hearts. Representative LVDP (A) and LVEDP (B) tracings are shown from 3W control and RIC groups. Left ventricular developed pressure (LVDP) is expressed as a percentage of pre-ischemic baseline values and left ventricular end diastolic pressure (LVEDP) is expressed in mm Hg. Values are expressed as means ± S.E.M. An (*) denotes a statistically significant difference (P<0.05) compared to control groups after 60 minutes of reperfusion.

Interestingly we showed for the first time that delayed and chronic RIC delivered daily for nine days produces similar cardioprotective benefits as acute 1W RIC (figure 4 and 5 A, B). LVDP was significantly greater (P<0.05) in both 2W and 3W RIC mice compared to the untreated controls (2W RIC – 87.5±5.5% vs. 63.2±8.2% and 3W RIC - 93.6±4.1% vs. 62. 3±6.0%) after global ischemia and 60 minutes of reperfusion. Furthermore, we also observed a significant attenuation of post-infarct diastolic pressures after delayed and chronic preconditioning compared to controls (2W – 154±2.6 mmHg vs. 38.4±5.4 mmHg and 3W RIC - 16. 9±2.8 mmHg vs. 40.1±5.6 mmHg) at the end of reperfusion.

### RIC enhances autophagy-signaling pathways

A main objective of the study was to examine the effects of acute, delayed and chronic RIC on components of cellular autophagy signaling pathways, including AMPK, mTOR, beclin-1, Atg5, LC3II/I, cathepsin L and p62. Using western blot analysis, we assessed the protein expression of each signaling component in each modality of preconditioning.

**Figure 6:**
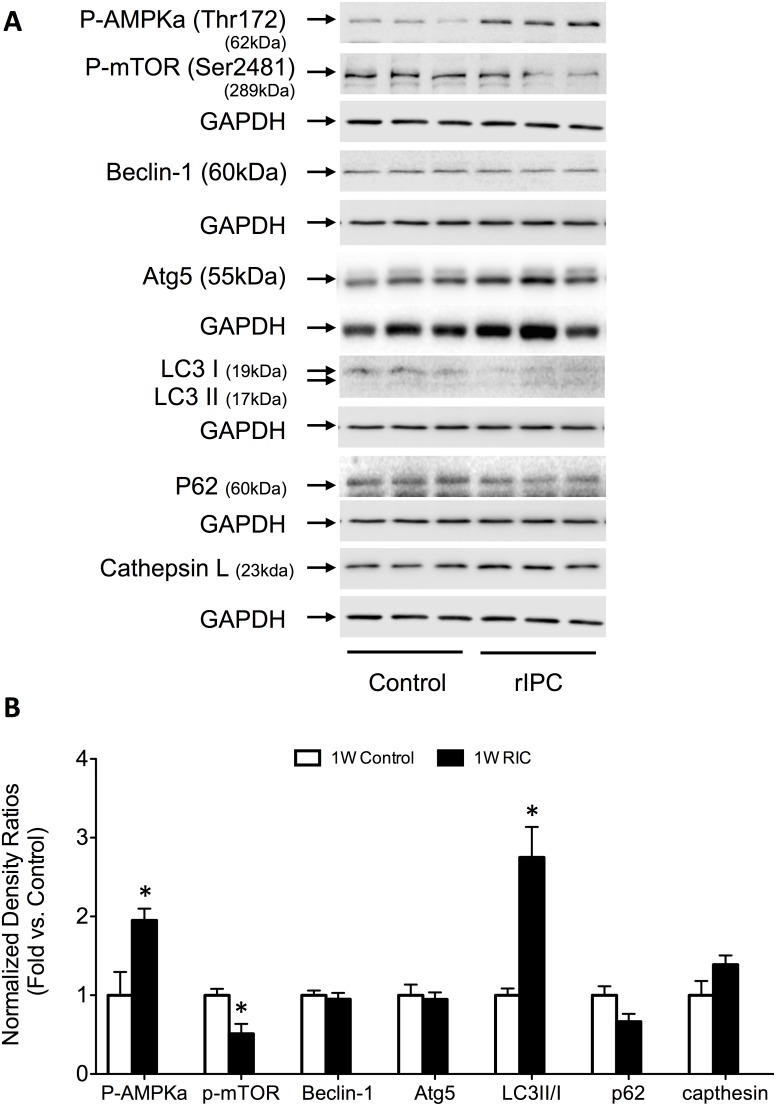
Acute, first window RIC activates autophagy signaling via p-AMPK upregulation and concomitant downregulation of mTOR. (A) Western blots for autophagy related signaling proteins. (B) Quantification of the protein fold change in 1W RIC compared to 1W controls. Values are means ± S.E.M. n = 6–8 per group. An (*) denotes a statistically significant difference (P<0.05) compared to control. (P-: phospho-).

Acute conditioning induced a significant (P<0.05) increase in p-AMPK levels – a positive regulator of autophagy activated during energy depletion - (1.9±0.1 – fold increase compared to control) and a concomitant significant decrease in the phosphorylated and activated form of its downstream target molecule, mTOR – a Ser/Thr kinase and negative regulator of autophagy (0.5±0.1 – fold decrease compared to control) (figure 6A, B). In addition we observed nearly a three-fold increase (P<0.05) in microtubule associated protein light chain 3, LC3II/I ratio in 1W RIC mice – a critical component of autophagosome membrane formation (2.7±0.4 – fold increase compared to controls). There were no significant differences observed in the other components of autophagy between 1W RIC and control groups.

**Figure 7:**
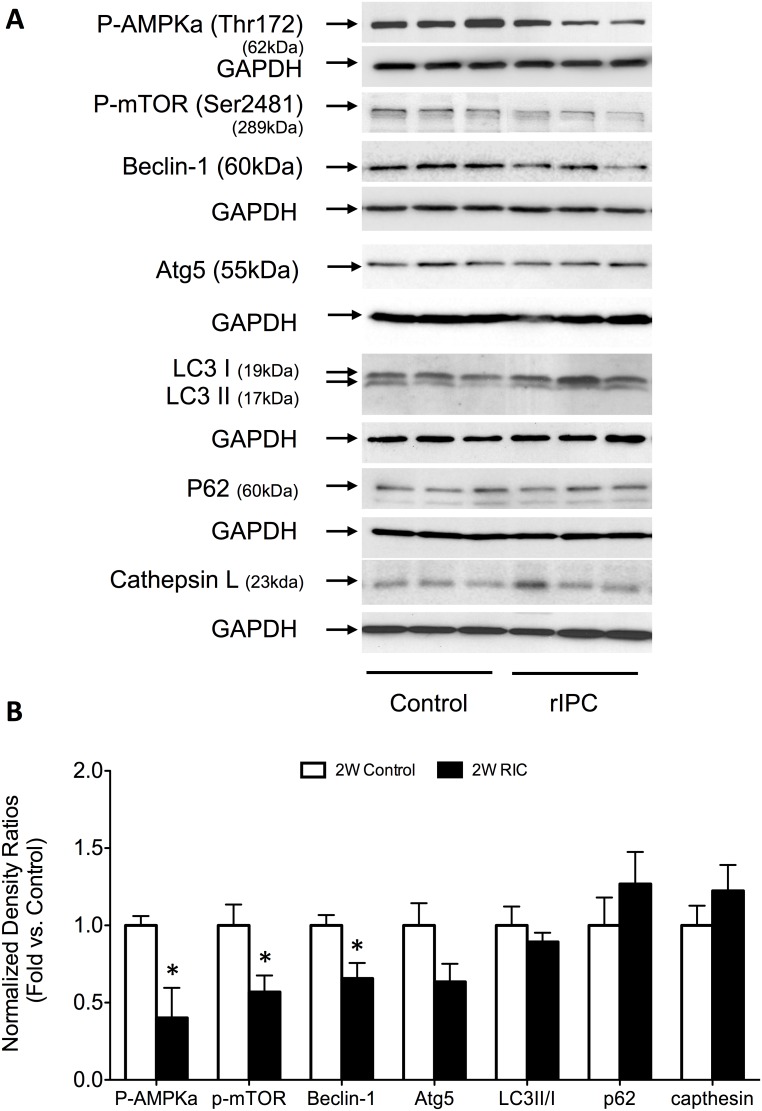
Delayed, second window RIC maintains mTOR inhibition without activating autophagosome machinery. (A) Western blots for autophagy related signaling proteins. (B) Quantification of the protein fold change in 2W RIC compared to 2W controls. Values are means ± S.E.M. n = 6–8 per group. An (*) denotes a statistically significant difference (P<0.05) compared to control. (P-: phospho-).

During delayed preconditioning the acute activation of AMPK is lost with a significant decrease (P<0.05) in its phosphorylated protein expression compared to 2W control mice (0.4±0.2 – fold decrease compared to controls). Interestingly, the inhibition of mTOR is sustained during delayed preconditioning (0.6±0.1 – fold decrease compared to control) (P<0.05) (figure 7A, B). However, we found that autophagy signaling may not be a predominant feature of delayed/second window preconditioning. This is highlighted with a significant (P<0.05) decrease in the expression of beclin-1, a cellular complex important for initiating autophagy (P<0.05) in 2W RIC mice (0.6±0.1 – fold decrease compared to controls). Other proteins involved in autophagy showed no significant differences between 2W RIC and control groups.

**Figure 8:**
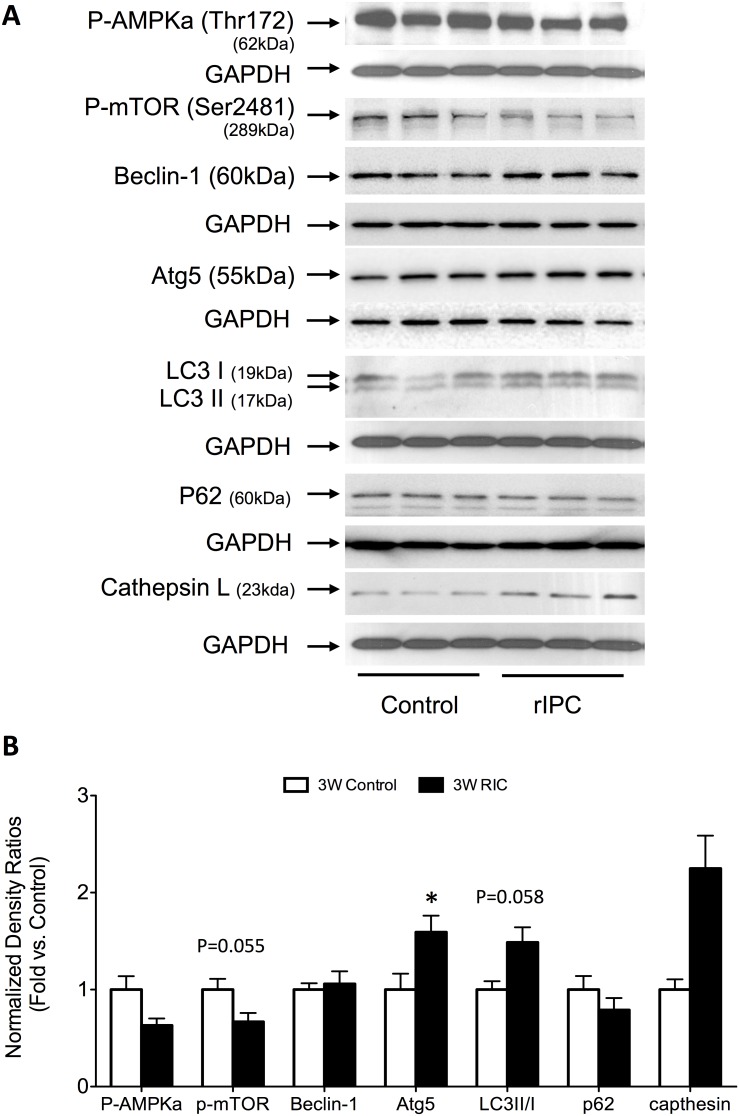
Chronic, third window RIC increases the expression of autophagosome proteins, LC3I/II and Atg5. (A) Western blots for autophagy related signaling proteins. (B) Quantification of the protein fold change in 3W RIC compared to 3W controls. Values are means ± S.E.M. n = 6–8 per group. An (*) denotes a statistically significant difference (P<0.05) compared to control. (P-: phospho-).

Similar to both acute and delayed preconditioning, we found that phosphorylated-mTOR expression remains decreased in 3W mice (0.7±0.1 – fold decrease compared to control, P = 0.055). This inhibition is likely achieved through a non-AMPK mechanism, as AMPK kinase levels trended towards decreased activation (0.6±0.1 – fold decrease compared to controls). However, unlike delayed preconditioning, it appears that the autophagy machinery is reactivated following chronic stimulation. We observed increases in the levels proteins associated with autophagy signaling, including LC3II/I (1.4±0.2 – fold increase compared to control) (P = 0.0587), Atg5 – an important autophagy-related protein (1.6±0.2 – fold increase compared to control) (P<0.05) and cathepsin L, (2.2±0.3 – fold increase compared to control) (P<0.05) in 3W RIC. P62, a sensor of proteotoxicity consumed by the active autophagosome, was unchanged during each preconditioning modality (figure 8A, B).

### Kinase responses in second window and chronic RIC

**Figure 9:**
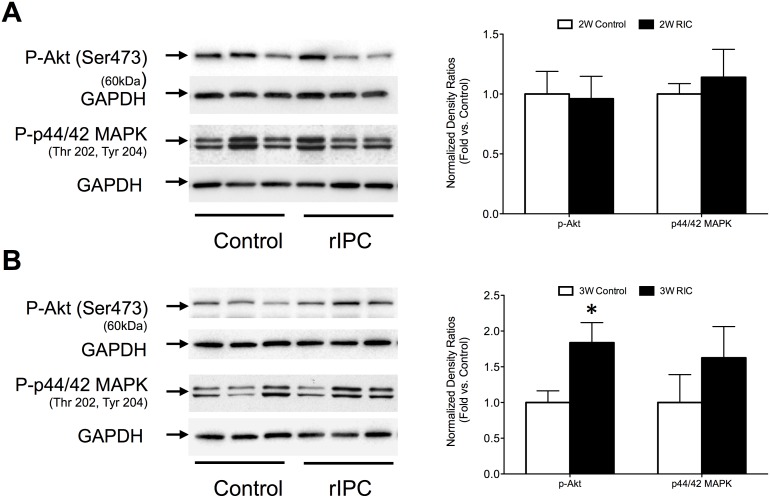
Chronic RIC activates kinase signaling in mouse myocardium. Western blots for phospho-Akt and phospho-Erk (p44/42 MAPK) in 2W(A) and 3W (B) RIC and control groups. Quantification of the protein fold change is shown in the graph to the right of blots. Values are means ± S.E.M. n = 6–8 per group. An (*) denotes a statistically significant difference (P<0.05) compared to control. (P-: phospho-).

We have previously reported that RIC acutely induces a portfolio of protective intracellular kinase signaling (e.g. PKCε, Akt, Erk), similar to that seen in local IC. The role of kinase signaling in delayed RIC responses appears much less significant. We found no significant differences in the expression of phosphorylated-Akt and phosphorylated-Erk in 2W RIC mice. Chronic preconditioning was associated with a small but significant (P<0.05) increase in the expression of p-Akt (1.8±0.3 – fold increase compared to control), but there was no significant change in p-Erk expression. (figure 9A, B).

## Discussion

To our knowledge, this is the first study to examine the effects of acute, delayed and chronic RIC on autophagy signaling in the myocardium. Our data show that repeated RIC leads to downregulation of mTOR and subsequent upregulation of pro-autophagy proteins, and is associated with levels of cardioprotection similar to that seen with the acute stimulus.

### Delayed and chronic RIC reduce infarction and improve cardiac functional recovery

The acute phase of cardioprotection induced by either local or remote ischemic preconditioning is associated with a well-established portfolio of intracellular kinase responses, which function to prevent mitochondrial-induced cell death. The delayed and chronic mechanisms of preconditioning similarly promote cell survival but have been suggested to do so primarily through changes in gene transcription and *de novo* protein synthesis that modify both the local and systemic responses (e.g. inflammation) to IR injury. The delayed response to RIC, while documented to be present in several organs, has been relatively sparsely studied in experimental and clinical studies of cardioprotection [19]–[22]. However, in a clinical trial of children undergoing cardiac surgery, delayed RIC was shown to reduce biomarkers of cardiac injury, and modify the inflammatory response [23]. A very recent experimental study in a rat model found that delayed RIC provided additional cardioprotection over and above that seen with acute sevoflurane-induced cardioprotection, via enhanced HO-1 expression via Nrf2 translocation [24]. There are no prior data examining the myocardial effects of repeated RIC, delivered for daily for 10 days (as in our present studies of ‘chronic’ cardioprotection), although we have previously demonstrated that such a regime induces sustained reduction in human neutrophil adhesiveness and phagocytotic activity, which may themselves play a role in modulating responses to IR injury [25]. For this reason, we chose to study the cardioprotective effect of this regime in an ex-vivo Langendorff model of IR injury, to avoid potential confounding effects. Consequently, we showed that delayed and chronic RIC preserve post-ischemic cardiac function to a similar degree as acute RIC, with improvements in both post-ischemic LVDP and LVEDP, but that the underlying patterns of kinase and autophagy signaling differs.

### The role of autophagy signaling in cardioprotection against IR injury

Autophagy has been identified as a pro-survival strategy utilized by cardiomyocytes during IR injury to prevent protein aggregation and cell death. Ischemia-reperfusion injury results in a battery of biochemical, metabolic and structural changes in the heart. At the onset of ischemia, oxidative phosphorylation is arrested resulting in an abrupt decline in ATP levels and eventually a breakdown of cell homeostasis [1]. The ensuing change in ion distribution across the cell and the accumulation of reactive oxygen species (ROS) damages the cells protein machinery [26]. Restoration of flow during reperfusion exacerbates these response and leads to cell dysfunction and eventual death. Activation of autophagy can help to mitigate the damage of IR injury and support cell function through the clearing damaged protein aggregates, removal of damaged ROS-producing mitochondria and through the recycling of macromolecules for use in cell repair.

Several studies have highlighted the benefits of autophagy during the acute and chronic phases of ischemic injury. For instance, it was found that treatment with autophagosome-lysosomal fusion inhibitor, bafilomycin A1, significantly aggravated post-infarction adverse remodeling in a mouse model of in-vivo IR injury [27]. In the same study it was found that treatment with rapamycin, an inhibitor of mTOR and a pro-autophagic stimulant was associated with improved functional and histological outcomes during the sub-acute and chronic post-infarct phases [27].

It has been suggested that cardioprotective strategies, such as ischemic preconditioning, can enhance autophagy to protect the myocardium from the deleterious effects of IR injury and its associated long-term sequalae [15], [28]. While this concept has already been demonstrated in investigations of local IPC, the present study is the first to show that RIC can also augment autophagy as part of its cardioprotective repertoire. Preconditioning may activate the autophagic machinery through one of two mechanisms, which may be differentially activated during the early and delayed/chronic phases. These include (1) PKC- or rising AMP- induced AMPK activation and subsequent mTOR inhibition or (2) Beclin-1 class III PI3K complex formation.

### RIC and autophagy

In the present study we show that factors involved in initiating autophagy and subsequent assembly of the autophagosome are induced as early as the first window of remote preconditioning. Within 1 hour of the RIC stimulus, there was a significant rise in the level of phosphorylated-AMPK, inhibition of the downstream regulator mTOR, and increases in LC3II/I, key regulators involved in autophagosome assembly. Increased AMPK during local ischemia has previously been implicated as an important mechanism in cytosolic ATP maintenance [29]. It is ubiquitously expressed in metabolically active tissues, such as skeletal and cardiac muscle, functioning as an intracellular fuel sensor that is activated upon depletion of energy stores [30]. During IR injury, intracellular stores of ATP are rapidly utilized and are not replenished with decreasing glucose supply. Depleting ATP levels give rise to increasing cytoplasmic adenosine monophosphate (AMP), an end product of ATP utilization and a potent signal for AMPK [31].

It is interesting that remote ischemia induces a similar response in AMPK signaling, even though the myocardium itself is not rendered ischemic. However, activation of AMPK has been previously implicated in the cardioprotective effects of IPC through a PKC-induced AMPK up-regulation of glucose transporters at the cell membrane [32]. In addition to enhancing mechanisms that maintain ATP production, AMPK serves as a master regulator of the autophagy pathway through deactivation of mTOR, itself a negative regulator of autophagy [28]. Interestingly, while we showed in the present study that RIC makes use of AMPK in the early phases of protection, its up-regulation does not appear to be a feature of the delayed and chronic phases. This observation fits with our current understanding of the later phases of RIC. Acute RIC is mediated primarily through activation of the reperfusion injury salvage kinases (RISK), which include the PI3k-Akt-PKC-Erk signaling cascade [33]. Therefore it is possible that the acute kinase response involving PKC leads to AMPK activation through a non-ATP depleting mechanism.

While initiation of autophagic mechanisms occurs during the acute phase of preconditioning, there is prior evidence suggesting it is primarily a phenomenon of long-term cardioprotection. Indeed, autophagy has previously implicated as a factor of local cardioprotection in the chronically preconditioned myocardium. A study by Yan et al. examining the effects of repetitive coronary stenosis in pigs found a marked increase in autophagic protein expression (cathepsin B and D, Beclin 1 and LC3-II) after six episodes of stenosis delivered 12 hourly over 3 days, compared to just one [34]. Further studies by the same group confirmed local conditioning to lead to up-regulation of autophagy with increases in the transcription of autophagy-related proteins [17], [18].

In the present study, we showed that the early significant increase in kinase signaling is substantially lost after ten days of repeated limb ischemia, and there is up-regulation of the expression of LC3-II and cathepsin L (a lysosomal cysteine protease involved in autophagy signaling). Additionally, we found a significant increase in autophagic machinery protein Atg5, a rate-limiting protein involved in autophagosome formation, in chronically preconditioned mice. LC3-II and Atg5 are both critical for the final maturation of the autophagosome [35] and their increase after repeated transient ischemia provides further support that autophagy may be a treatment paradigm of long term conditioning. In a human study examining the effects of RIC in patients undergoing coronary artery bypass grafting (CABG), it was found that only LC3II/I was elevated at reperfusion, although other markers of autophagy signaling (Atg5) were unaffected [36]. These findings are consistent with those generated in this study in which we found that not all the autophagosomal components are fully formed during the acute phase of preconditioning. That said, the mechanisms underlying how chronic RIC augments the autophagic machinery remain unclear. It is possible that activation occurs through a beclin-1 mediated process similar to local IPC. However, our results show only a slight increase in beclin-1 expression after chronic RIC. Interestingly we showed that mTOR inhibition is sustained during both the delayed and chronic phases of RIC, the latter without a concomitant increase in AMPK, suggesting that other signaling pathways involved with the preconditioning phenotype may be operating to limit its function. Our data therefore provide proof-of-principle upon which future investigations should be based.

Another measure of autophagic flux in addition to LC3-II and Atg5 is the level of p62 within the cell. As previously discussed, p62 is an adaptor protein that binds to ubiquinated protein products destined for consumption by the autophagsome machinery. As such, accumulation of p62 can be used as a marker of proteotoxicty buildup within the cell and a decrease in the function of autophagic clearance mechanisms [26]. Conversely, low levels of p62 imply active intracellular phagocytsosis. However, given that RIC is a non-lethal stimulus, there is likely no build up of protein aggregates, which may provide some explanation for the low levels of p62 seen during each phase of conditioning examined in this study.

While the underlying mechanisms remain to be elucidated in future studies, there are important implications of our data regarding chronic autophagy signaling. For example, inhibition of mTOR signaling has been shown to be beneficial for remodeling of the post-infarcted myocardium. In a study by Buss et al., it was shown that 28 days of mTOR inhibition using everolimus following experimental myocardial infarction was associated with improvements in LV function and end-diastolic diameters [37]. This was also associated with reduced infarction and an increase in cellular autophagy mechanisms in the injury zone. The authors concluded that inhibition of mTOR-mediated protein synthesis was critical for preventing pathological LV hypertrophy. The long-term activation of autophagy with mTOR inhibition and its effects on minimizing adverse LV remodeling is consistent with a previous study conducted by our group in which we showed similar benefits with chronic RIC for 28 days after MI in a rat model [14]. While autophagy wasn’t directly examined in that study, our current the findings showing chronically conditioned myocardium induces upregulation of autophagy may provide a mechanistic explanation.

Another interesting finding generated from this study involves a potential interaction between autophagy related proteins and elements of the RISK pathway. We observed that chronic RIC recapitulated elements of the RISK pathway that are active components in the acute phases of protection. Specifically, we found an increase in the levels of Akt after nine days of remote conditioning. This finding suggests the growing hypothesis that chronic RIC utilizes multiple signaling mechanisms to generate the cardioprotective phenotype [18]. Indeed, recently Martinez-Lopez et al. has shown that the cell proliferation regulator, extracellular signal regulated kinase (ERK), which is an important component of the RISK pathway of preconditioning, localizes with autophagy proteins (ATG) [38]. Furthermore, their study shows that deletion of Atg5 results in decreased ERK activity. Our results suggest that a similar interaction may exist with, the serine threonine kinase, Akt. The interactions between RISK signaling and autophagy proteins and their relation to the cardioprotective effects of preconditioning warrant further study to better understand the mechanisms various mechanisms at work in the chronically conditioned heart.

There are some limitations to the current study. Firstly, it important to note that while the current findings are the first to examine the existence of autophagy signaling in the cardioprotection of RIC, we did not examine the functional significance of each signal. We have generated a unique set of preliminary observations, which implicate autophagy in the cellular mechanisms of preconditioning for future studies. These may involve investigating the effects of upregulation or inhibition of autophagy on the cardioprotection granted by RIC. Secondly, the findings generated in this study are cardiac specific and may not apply in other organs (e.g. brain, liver kidney), particularly with respect to mTOR activation. For example, a study examining the effects of RIC on the hippocampus after bilateral carotid artery occlusion found that an increase in mTOR activation was associated with neural cell protection [39]. Furthermore, inhibiting mTOR with rapamycin treatment blocked the effects of RIC. These incongruent findings have also been shown in studies of cardioprotection after RIC. In a study of local ischemic preconditioning it was found that mTOR is activated through the actions of Erk-P70S6K. It appears that preconditioning can operate via two available RISK pathways, the PI3K-Akt or the Erk-P70S6k-mTOR pathway [16]. Interestingly both pathways exhibit cross talk in that increased activation of one pathway leads to inhibition of other RISK signaling cascades. In the present study, it appears that RIC predominantly activates a PI3K-Akt pathway, which, in addition to AMPK activation, leads to an overall decrease in mTOR activation, permitting the initiation of autophagy.

In conclusion, we showed for the first time that a similar level of cardioprotection following transient ischemia exists in both the acute and delayed phases of preconditioning. Furthermore, chronically preconditioned mice exhibit cardioprotection against both tissue damage and ventricular dysfunction after ischemia-reperfusion injury similar to that observed with an isolated stimulus. In addition to traditional kinase signaling pathways (e.g. RISK), we identified a potential role for autophagy in the cellular mechanism of preconditioning, which may have broader implications in the protection against post-infarction left-ventricular remodeling and dysfunction.
